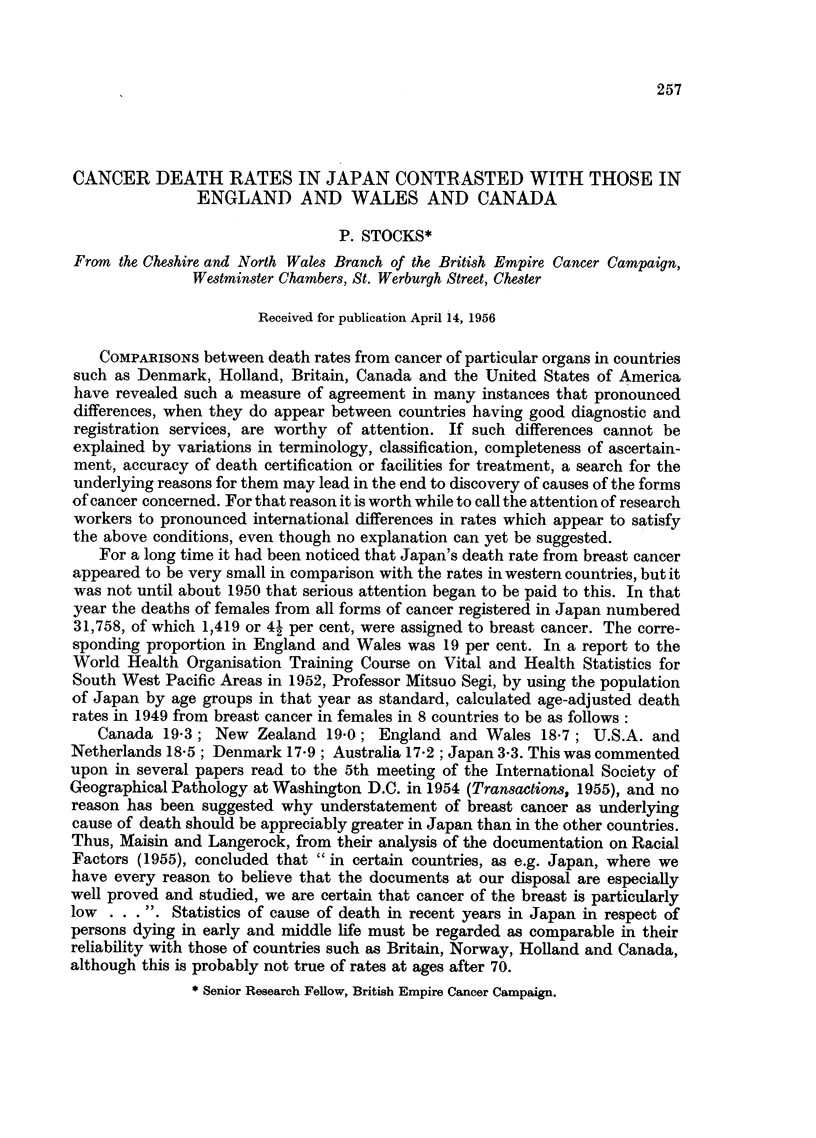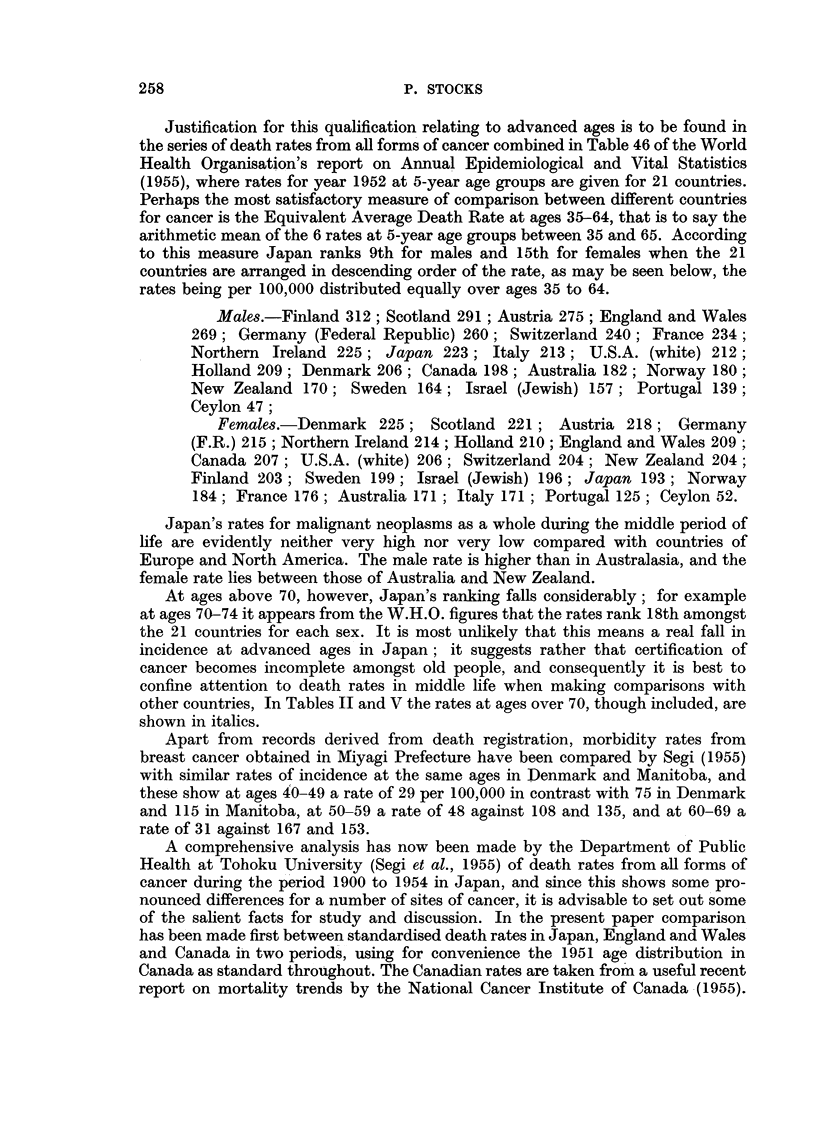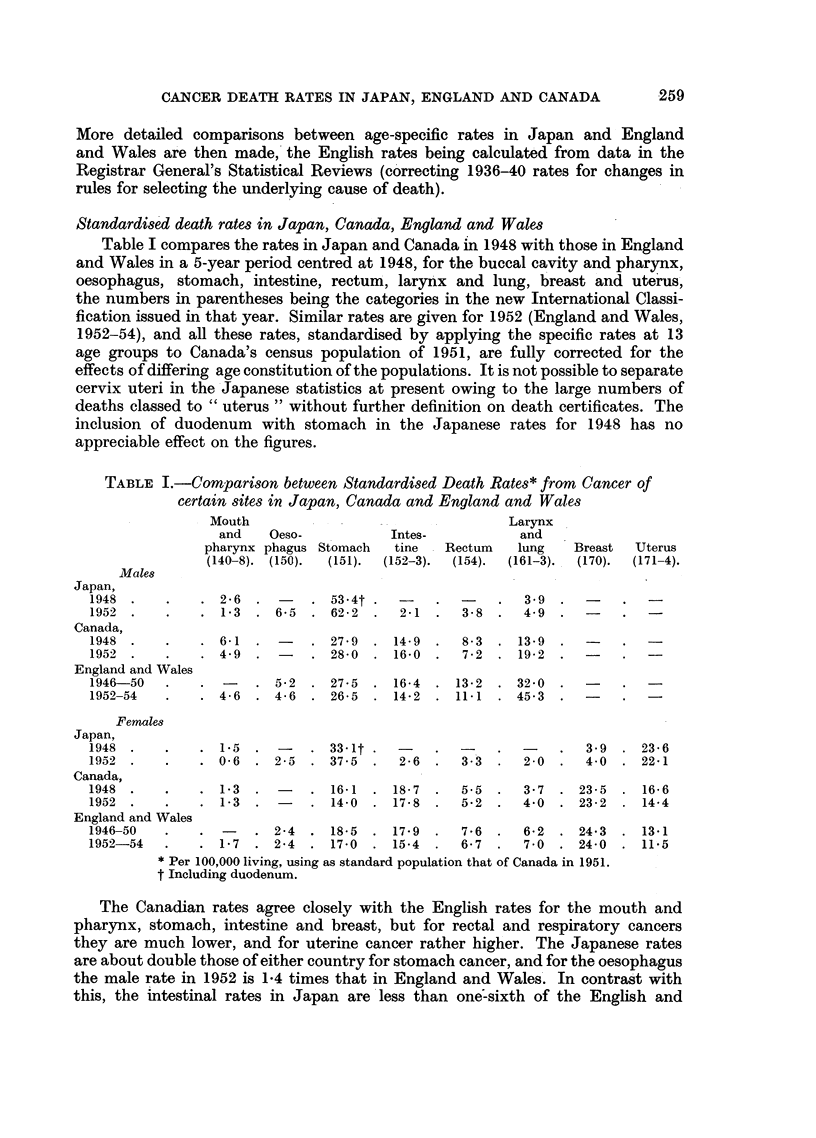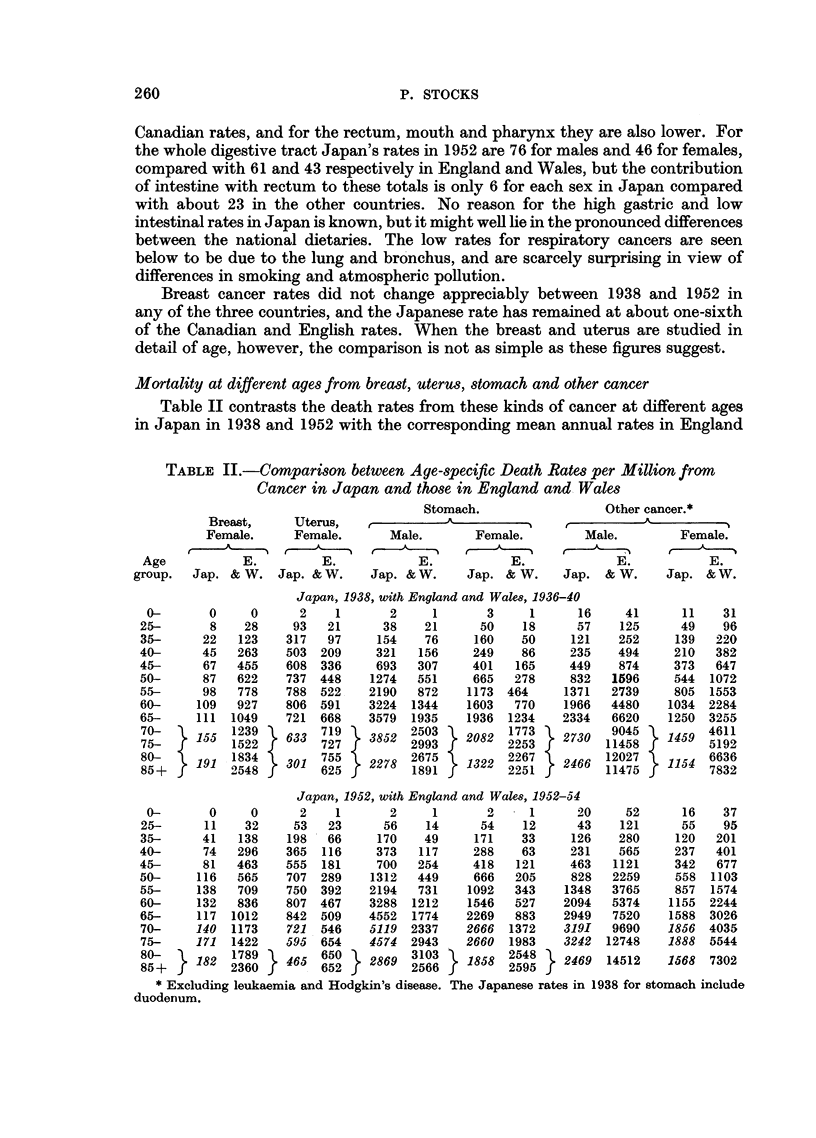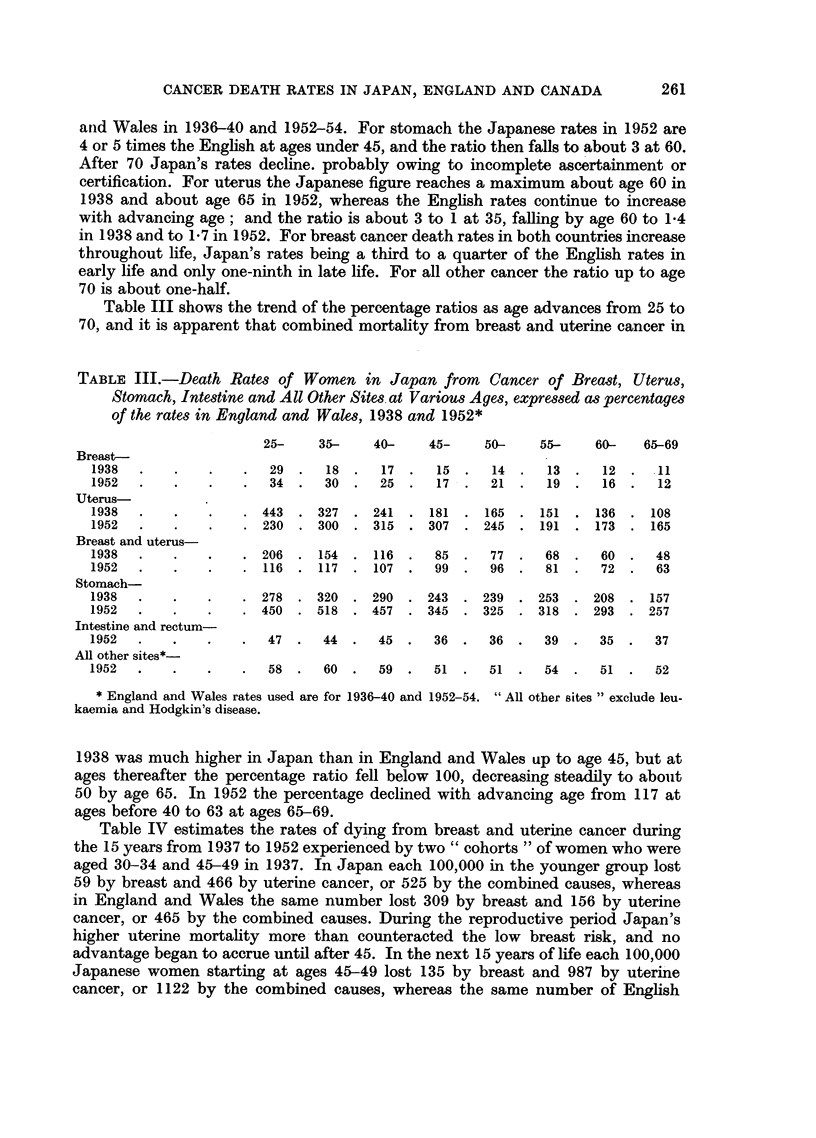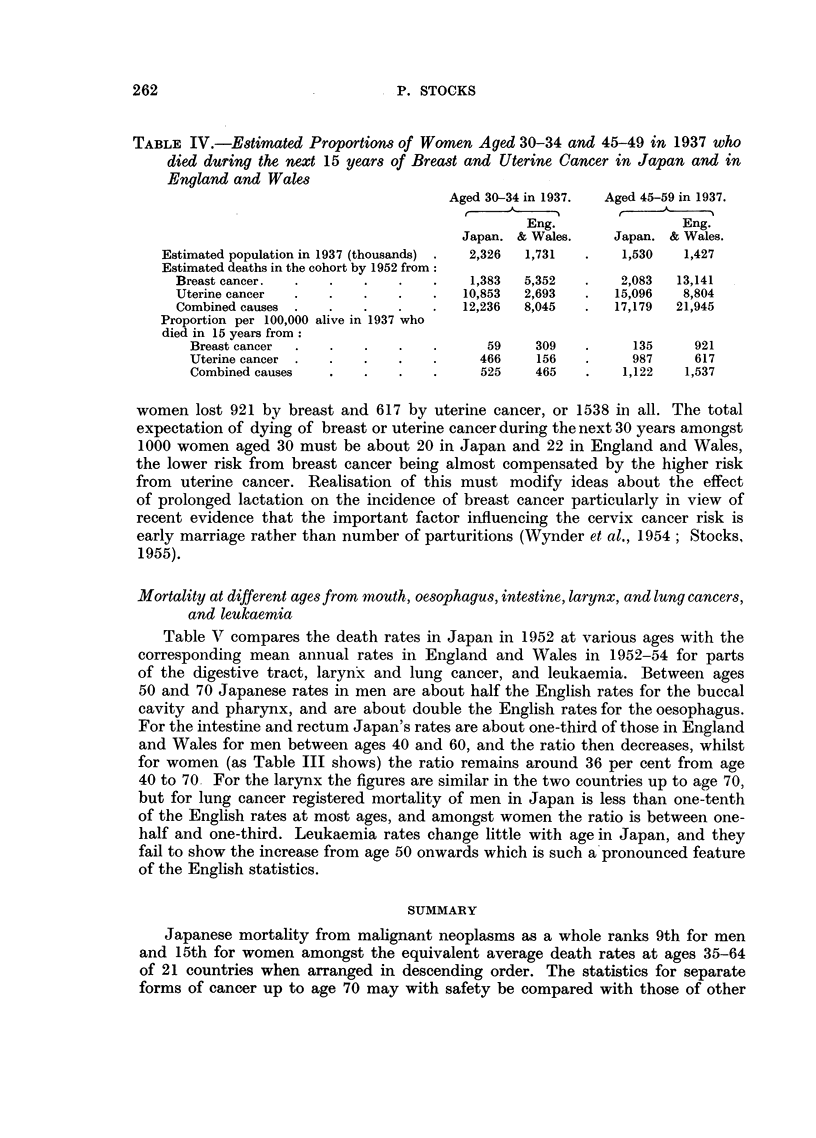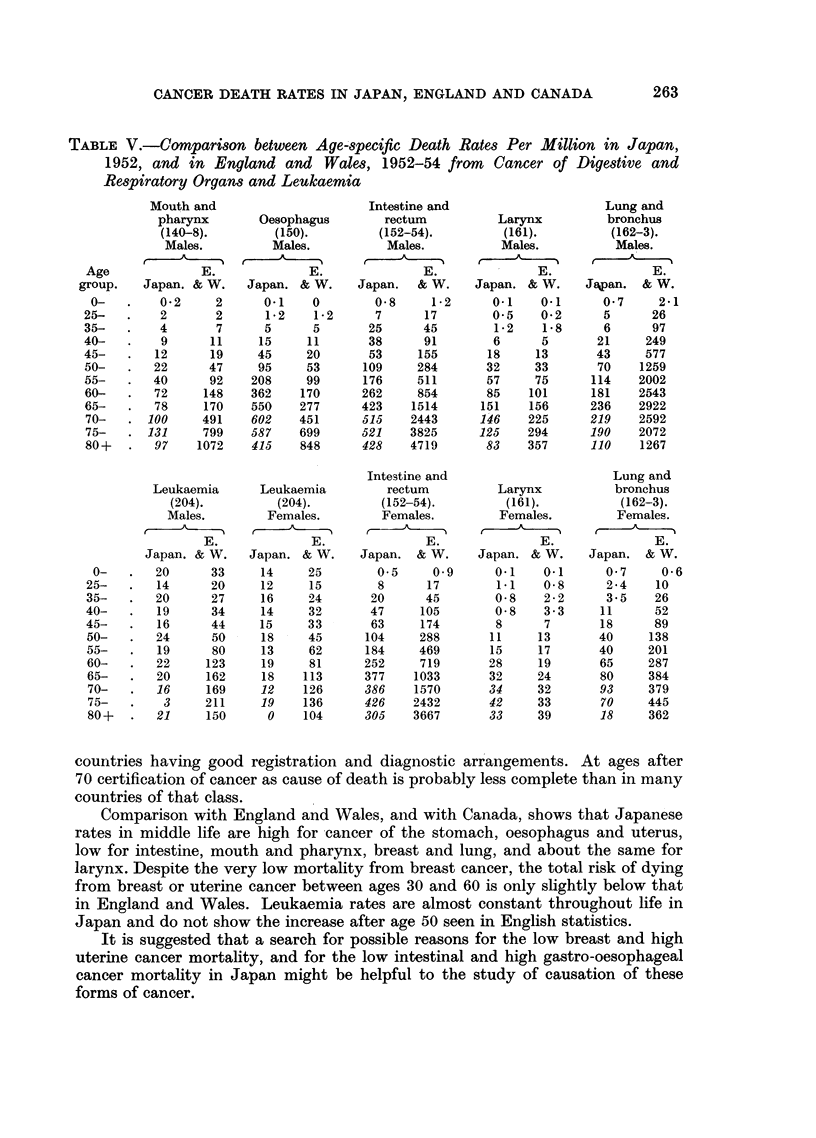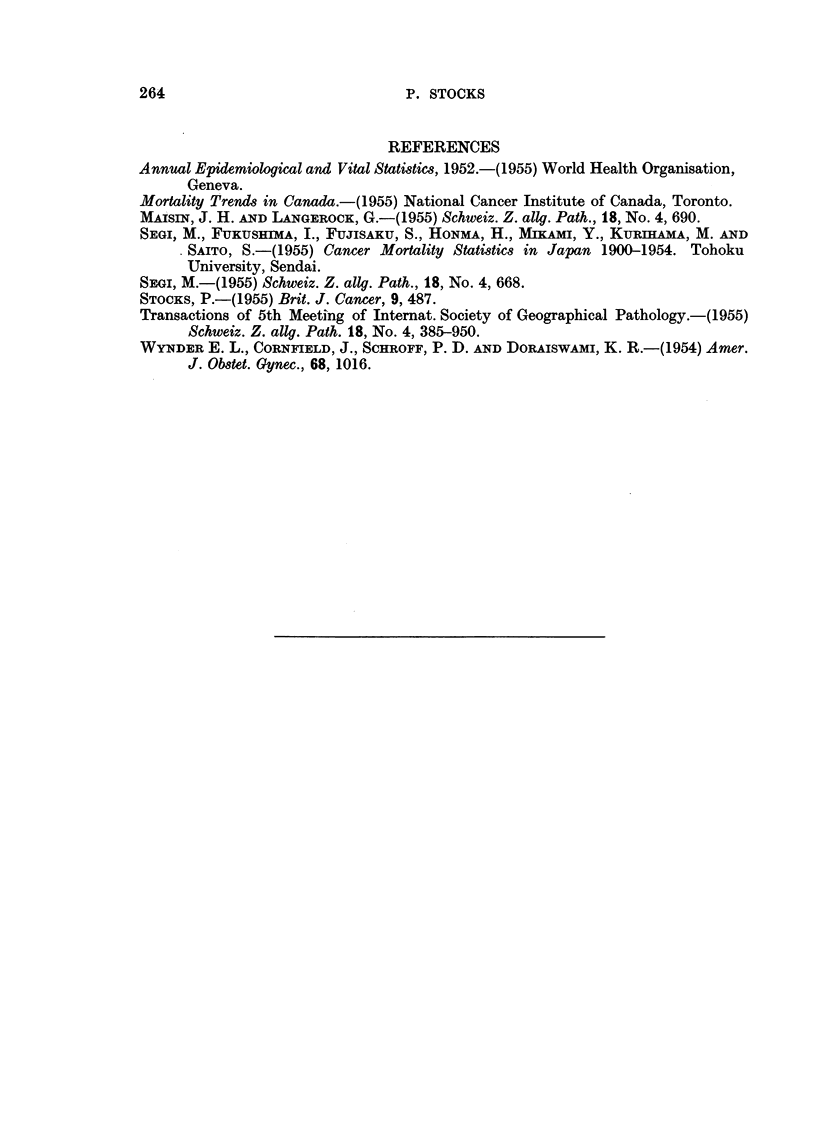# Cancer Death Rates in Japan Contrasted with those in England and Wales and Canada

**DOI:** 10.1038/bjc.1956.30

**Published:** 1956-06

**Authors:** P. Stocks


### CANCER DEATH RATES IN JAPAN CONTRASTED WITH THOSE IN ENGLAND AND WALES AND CANADA

P. STOCKS*

*From the Cheshire and North Wales Branch of the British Empire Cancer Campaign, Westminster Chambers, St. Werburgh Street, Chester*

Received for publication April 14, 1956

COMPARISONS between death rates from cancer of particular organs in countries such as Denmark, Holland, Britain, Canada and the United States of America have revealed such a measure of agreement in many instances that pronounced differences, when they do appear between countries having good diagnostic and registration services, are worthy of attention. If such differences cannot be explained by variations in terminology, classification, completeness of ascertainment, accuracy of death certification or facilities for treatment, a search for the underlying reasons for them may lead in the end to discovery of causes of the forms of cancer concerned. For that reason it is worth while to call the attention of research workers to pronounced international differences in rates which appear to satisfy the above conditions, even though no explanation can yet be suggested.

For a long time it had been noticed that Japan's death rate from breast cancer appeared to be very small in comparison with the rates in western countries, but it was not until about 1950 that serious attention began to be paid to this. In that year the deaths of females from all forms of cancer registered in Japan numbered 31,758, of which 1,419 or $4\frac{1}{2}$ per cent, were assigned to breast cancer. The corresponding proportion in England and Wales was 19 per cent. In a report to the World Health Organisation Training Course on Vital and Health Statistics for South West Pacific Areas in 1952, Professor Mitsuo Segi, by using the population of Japan by age groups in that year as standard, calculated age-adjusted death rates in 1949 from breast cancer in females in 8 countries to be as follows :

Canada 19·3 ; New Zealand 19·0 ; England and Wales 18·7 ; U.S.A. and Netherlands 18·5 ; Denmark 17·9 ; Australia 17·2 ; Japan 3·3. This was commented upon in several papers read to the 5th meeting of the International Society of Geographical Pathology at Washington D.C. in 1954 (*Transactions,* 1955), and no reason has been suggested why understatement of breast cancer as underlying cause of death should be appreciably greater in Japan than in the other countries. Thus, Maisin and Langerock, from their analysis of the documentation on Racial Factors (1955), concluded that " in certain countries, as e.g. Japan, where we have every reason to believe that the documents at our disposal are especially well proved and studied, we are certain that cancer of the breast is particularly low . . . ". Statistics of cause of death in recent years in Japan in respect of persons dying in early and middle life must be regarded as comparable in their reliability with those of countries such as Britain, Norway, Holland and Canada, although this is probably not true of rates at ages after 70.

* Senior Research Fellow, British Empire Cancer Campaign.

Justification for this qualification relating to advanced ages is to be found in the series of death rates from all forms of cancer combined in Table 46 of the World Health Organisation's report on Annual Epidemiological and Vital Statistics (1955), where rates for year 1952 at 5-year age groups are given for 21 countries. Perhaps the most satisfactory measure of comparison between different countries for cancer is the Equivalent Average Death Rate at ages 35–64, that is to say the arithmetic mean of the 6 rates at 5-year age groups between 35 and 65. According to this measure Japan ranks 9th for males and 15th for females when the 21 countries are arranged in descending order of the rate, as may be seen below, the rates being per 100,000 distributed equally over ages 35 to 64.

*Males.*—Finland 312 ; Scotland 291 ; Austria 275 ; England and Wales 269 ; Germany (Federal Republic) 260 ; Switzerland 240 ; France 234 ; Northern Ireland 225 ; *Japan* 223 ; Italy 213 ; U.S.A. (white) 212 ; Holland 209 ; Denmark 206 ; Canada 198 ; Australia 182 ; Norway 180 ; New Zealand 170 ; Sweden 164 ; Israel (Jewish) 157 ; Portugal 139 ; Ceylon 47 ;

*Females.*—Denmark 225 ; Scotland 221 ; Austria 218 ; Germany (F.R.) 215 ; Northern Ireland 214 ; Holland 210 ; England and Wales 209 ; Canada 207 ; U.S.A. (white) 206 ; Switzerland 204 ; New Zealand 204 ; Finland 203 ; Sweden 199 ; Israel (Jewish) 196 ; *Japan* 193 ; Norway 184 ; France 176 ; Australia 171 ; Italy 171 ; Portugal 125 ; Ceylon 52.

Japan's rates for malignant neoplasms as a whole during the middle period of life are evidently neither very high nor very low compared with countries of Europe and North America. The male rate is higher than in Australasia, and the female rate lies between those of Australia and New Zealand.

At ages above 70, however, Japan's ranking falls considerably ; for example at ages 70–74 it appears from the W.H.O. figures that the rates rank 18th amongst the 21 countries for each sex. It is most unlikely that this means a real fall in incidence at advanced ages in Japan ; it suggests rather that certification of cancer becomes incomplete amongst old people, and consequently it is best to confine attention to death rates in middle life when making comparisons with other countries, In Tables II and V the rates at ages over 70, though included, are shown in italics.

Apart from records derived from death registration, morbidity rates from breast cancer obtained in Miyagi Prefecture have been compared by Segi (1955) with similar rates of incidence at the same ages in Denmark and Manitoba, and these show at ages 40–49 a rate of 29 per 100,000 in contrast with 75 in Denmark and 115 in Manitoba, at 50–59 a rate of 48 against 108 and 135, and at 60–69 a rate of 31 against 167 and 153.

A comprehensive analysis has now been made by the Department of Public Health at Tohoku University (Segi *et al.*, 1955) of death rates from all forms of cancer during the period 1900 to 1954 in Japan, and since this shows some pronounced differences for a number of sites of cancer, it is advisable to set out some of the salient facts for study and discussion. In the present paper comparison has been made first between standardised death rates in Japan, England and Wales and Canada in two periods, using for convenience the 1951 age distribution in Canada as standard throughout. The Canadian rates are taken from a useful recent report on mortality trends by the National Cancer Institute of Canada (1955).

More detailed comparisons between age-specific rates in Japan and England and Wales are then made, the English rates being calculated from data in the Registrar General's Statistical Reviews (correcting 1936–40 rates for changes in rules for selecting the underlying cause of death).

*Standardised death rates in Japan, Canada, England and Wales*

Table I compares the rates in Japan and Canada in 1948 with those in England and Wales in a 5-year period centred at 1948, for the buccal cavity and pharynx, oesophagus, stomach, intestine, rectum, larynx and lung, breast and uterus, the numbers in parentheses being the categories in the new International Classification issued in that year. Similar rates are given for 1952 (England and Wales, 1952–54), and all these rates, standardised by applying the specific rates at 13 age groups to Canada's census population of 1951, are fully corrected for the effects of differing age constitution of the populations. It is not possible to separate cervix uteri in the Japanese statistics at present owing to the large numbers of deaths classed to " uterus " without further definition on death certificates. The inclusion of duodenum with stomach in the Japanese rates for 1948 has no appreciable effect on the figures.

TABLE I.—*Comparison between Standardised Death Rates* from Cancer of certain sites in Japan, Canada and England and Wales*

| | Mouth and pharynx (140–8). | Oeso-phagus (150). | Stomach (151). | Intes-tine (152–3). | Rectum (154). | Larynx and lung (161–3). | Breast (170). | Uterus (171–4). |
|---|---|---|---|---|---|---|---|---|
| *Males* | | | | | | | | |
| Japan, | | | | | | | | |
| 1948 | 2·6 | — | 53·4† | — | — | 3·9 | — | — |
| 1952 | 1·3 | 6·5 | 62·2 | 2·1 | 3·8 | 4·9 | — | — |
| Canada, | | | | | | | | |
| 1948 | 6·1 | — | 27·9 | 14·9 | 8·3 | 13·9 | — | — |
| 1952 | 4·9 | — | 28·0 | 16·0 | 7·2 | 19·2 | — | — |
| England and Wales | | | | | | | | |
| 1946—50 | — | 5·2 | 27·5 | 16·4 | 13·2 | 32·0 | — | — |
| 1952–54 | 4·6 | 4·6 | 26·5 | 14·2 | 11·1 | 45·3 | — | — |
| *Females* | | | | | | | | |
| Japan, | | | | | | | | |
| 1948 | 1·5 | — | 33·1† | — | — | — | 3·9 | 23·6 |
| 1952 | 0·6 | 2·5 | 37·5 | 2·6 | 3·3 | 2·0 | 4·0 | 22·1 |
| Canada, | | | | | | | | |
| 1948 | 1·3 | — | 16·1 | 18·7 | 5·5 | 3·7 | 23·5 | 16·6 |
| 1952 | 1·3 | — | 14·0 | 17·8 | 5·2 | 4·0 | 23·2 | 14·4 |
| England and Wales | | | | | | | | |
| 1946–50 | — | 2·4 | 18·5 | 17·9 | 7·6 | 6·2 | 24·3 | 13·1 |
| 1952—54 | 1·7 | 2·4 | 17·0 | 15·4 | 6·7 | 7·0 | 24·0 | 11·5 |

* Per 100,000 living, using as standard population that of Canada in 1951.
† Including duodenum.

The Canadian rates agree closely with the English rates for the mouth and pharynx, stomach, intestine and breast, but for rectal and respiratory cancers they are much lower, and for uterine cancer rather higher. The Japanese rates are about double those of either country for stomach cancer, and for the oesophagus the male rate in 1952 is 1·4 times that in England and Wales. In contrast with this, the intestinal rates in Japan are less than one-sixth of the English and

Canadian rates, and for the rectum, mouth and pharynx they are also lower. For the whole digestive tract Japan's rates in 1952 are 76 for males and 46 for females, compared with 61 and 43 respectively in England and Wales, but the contribution of intestine with rectum to these totals is only 6 for each sex in Japan compared with about 23 in the other countries. No reason for the high gastric and low intestinal rates in Japan is known, but it might well lie in the pronounced differences between the national dietaries. The low rates for respiratory cancers are seen below to be due to the lung and bronchus, and are scarcely surprising in view of differences in smoking and atmospheric pollution.

Breast cancer rates did not change appreciably between 1938 and 1952 in any of the three countries, and the Japanese rate has remained at about one-sixth of the Canadian and English rates. When the breast and uterus are studied in detail of age, however, the comparison is not as simple as these figures suggest.

*Mortality at different ages from breast, uterus, stomach and other cancer*

Table II contrasts the death rates from these kinds of cancer at different ages in Japan in 1938 and 1952 with the corresponding mean annual rates in England

TABLE II.—*Comparison between Age-specific Death Rates per Million from Cancer in Japan and those in England and Wales*

| | Breast, Female. | | Uterus, Female. | | Stomach. Male. | | Stomach. Female. | | Other cancer.* Male. | | Other cancer.* Female. | |
|---|---|---|---|---|---|---|---|---|---|---|---|---|
| Age group. | Jap. | E. & W. | Jap. | E. & W. | Jap. | E. & W. | Jap. | E. & W. | Jap. | E. & W. | Jap. | E. & W. |
| | *Japan, 1938, with England and Wales, 1936–40* | | | | | | | | | | | |
| 0– | 0 | 0 | 2 | 1 | 2 | 1 | 3 | 1 | 16 | 41 | 11 | 31 |
| 25– | 8 | 28 | 93 | 21 | 38 | 21 | 50 | 18 | 57 | 125 | 49 | 96 |
| 35– | 22 | 123 | 317 | 97 | 154 | 76 | 160 | 50 | 121 | 252 | 139 | 220 |
| 40– | 45 | 263 | 503 | 209 | 321 | 156 | 249 | 86 | 235 | 494 | 210 | 382 |
| 45– | 67 | 455 | 608 | 336 | 693 | 307 | 401 | 165 | 449 | 874 | 373 | 647 |
| 50– | 87 | 622 | 737 | 448 | 1274 | 551 | 665 | 278 | 832 | 1596 | 544 | 1072 |
| 55– | 98 | 778 | 788 | 522 | 2190 | 872 | 1173 | 464 | 1371 | 2739 | 805 | 1553 |
| 60– | 109 | 927 | 806 | 591 | 3224 | 1344 | 1603 | 770 | 1966 | 4480 | 1034 | 2284 |
| 65– | 111 | 1049 | 721 | 668 | 3579 | 1935 | 1936 | 1234 | 2334 | 6620 | 1250 | 3255 |
| 70– } | *155* | 1239 | *633* | 719 | *3852* | 2503 | *2082* | 1773 | *2730* | 9045 | *1459* | 4611 |
| 75– } | | 1522 | | 727 | | 2993 | | 2253 | | 11458 | | 5192 |
| 80– } | *191* | 1834 | *301* | 755 | *2278* | 2675 | *1322* | 2267 | *2466* | 12027 | *1154* | 6636 |
| 85+ } | | 2548 | | 625 | | 1891 | | 2251 | | 11475 | | 7832 |
| | *Japan, 1952, with England and Wales, 1952–54* | | | | | | | | | | | |
| 0– | 0 | 0 | 2 | 1 | 2 | 1 | 2 | 1 | 20 | 52 | 16 | 37 |
| 25– | 11 | 32 | 53 | 23 | 56 | 14 | 54 | 12 | 43 | 121 | 55 | 95 |
| 35– | 41 | 138 | 198 | 66 | 170 | 49 | 171 | 33 | 126 | 280 | 120 | 201 |
| 40– | 74 | 296 | 365 | 116 | 373 | 117 | 288 | 63 | 231 | 565 | 237 | 401 |
| 45– | 81 | 463 | 555 | 181 | 700 | 254 | 418 | 121 | 463 | 1121 | 342 | 677 |
| 50– | 116 | 565 | 707 | 289 | 1312 | 449 | 666 | 205 | 828 | 2259 | 558 | 1103 |
| 55– | 138 | 709 | 750 | 392 | 2194 | 731 | 1092 | 343 | 1348 | 3765 | 857 | 1574 |
| 60– | 132 | 836 | 807 | 467 | 3288 | 1212 | 1546 | 527 | 2094 | 5374 | 1155 | 2244 |
| 65– | 117 | 1012 | 842 | 509 | 4552 | 1774 | 2269 | 883 | 2949 | 7520 | 1588 | 3026 |
| 70– | *140* | 1173 | *721* | 546 | *5119* | 2337 | *2666* | 1372 | *3191* | 9690 | *1856* | 4035 |
| 75– | *171* | 1422 | *595* | 654 | *4574* | 2943 | *2660* | 1983 | *3242* | 12748 | *1888* | 5544 |
| 80– } | *182* | 1789 | *465* | 650 | *2869* | 3103 | *1858* | 2548 | *2469* | 14512 | *1568* | 7302 |
| 85+ } | | 2360 | | 652 | | 2566 | | 2595 | | | | |

* Excluding leukaemia and Hodgkin's disease. The Japanese rates in 1938 for stomach include duodenum.

and Wales in 1936–40 and 1952–54. For stomach the Japanese rates in 1952 are 4 or 5 times the English at ages under 45, and the ratio then falls to about 3 at 60. After 70 Japan's rates decline. probably owing to incomplete ascertainment or certification. For uterus the Japanese figure reaches a maximum about age 60 in 1938 and about age 65 in 1952, whereas the English rates continue to increase with advancing age ; and the ratio is about 3 to 1 at 35, falling by age 60 to 1·4 in 1938 and to 1·7 in 1952. For breast cancer death rates in both countries increase throughout life, Japan's rates being a third to a quarter of the English rates in early life and only one-ninth in late life. For all other cancer the ratio up to age 70 is about one-half.

Table III shows the trend of the percentage ratios as age advances from 25 to 70, and it is apparent that combined mortality from breast and uterine cancer in

TABLE III.—*Death Rates of Women in Japan from Cancer of Breast, Uterus, Stomach, Intestine and All Other Sites at Various Ages, expressed as percentages of the rates in England and Wales*, 1938 *and* 1952*

| | 25– | 35– | 40– | 45– | 50– | 55– | 60– | 65–69 |
|---|---|---|---|---|---|---|---|---|
| Breast— | | | | | | | | |
| 1938 | 29 | 18 | 17 | 15 | 14 | 13 | 12 | 11 |
| 1952 | 34 | 30 | 25 | 17 | 21 | 19 | 16 | 12 |
| Uterus— | | | | | | | | |
| 1938 | 443 | 327 | 241 | 181 | 165 | 151 | 136 | 108 |
| 1952 | 230 | 300 | 315 | 307 | 245 | 191 | 173 | 165 |
| Breast and uterus— | | | | | | | | |
| 1938 | 206 | 154 | 116 | 85 | 77 | 68 | 60 | 48 |
| 1952 | 116 | 117 | 107 | 99 | 96 | 81 | 72 | 63 |
| Stomach— | | | | | | | | |
| 1938 | 278 | 320 | 290 | 243 | 239 | 253 | 208 | 157 |
| 1952 | 450 | 518 | 457 | 345 | 325 | 318 | 293 | 257 |
| Intestine and rectum— | | | | | | | | |
| 1952 | 47 | 44 | 45 | 36 | 36 | 39 | 35 | 37 |
| All other sites*— | | | | | | | | |
| 1952 | 58 | 60 | 59 | 51 | 51 | 54 | 51 | 52 |

* England and Wales rates used are for 1936–40 and 1952–54. " All other sites " exclude leukaemia and Hodgkin's disease.

1938 was much higher in Japan than in England and Wales up to age 45, but at ages thereafter the percentage ratio fell below 100, decreasing steadily to about 50 by age 65. In 1952 the percentage declined with advancing age from 117 at ages before 40 to 63 at ages 65–69.

Table IV estimates the rates of dying from breast and uterine cancer during the 15 years from 1937 to 1952 experienced by two " cohorts " of women who were aged 30–34 and 45–49 in 1937. In Japan each 100,000 in the younger group lost 59 by breast and 466 by uterine cancer, or 525 by the combined causes, whereas in England and Wales the same number lost 309 by breast and 156 by uterine cancer, or 465 by the combined causes. During the reproductive period Japan's higher uterine mortality more than counteracted the low breast risk, and no advantage began to accrue until after 45. In the next 15 years of life each 100,000 Japanese women starting at ages 45–49 lost 135 by breast and 987 by uterine cancer, or 1122 by the combined causes, whereas the same number of English

TABLE IV.—*Estimated Proportions of Women Aged* 30–34 *and* 45–49 *in* 1937 *who died during the next* 15 *years of Breast and Uterine Cancer in Japan and in England and Wales*

| | Aged 30–34 in 1937. | | Aged 45–59 in 1937. | |
|---|---|---|---|---|
| | Japan. | Eng. & Wales. | Japan. | Eng. & Wales. |
| Estimated population in 1937 (thousands) | 2,326 | 1,731 | 1,530 | 1,427 |
| Estimated deaths in the cohort by 1952 from : | | | | |
| Breast cancer | 1,383 | 5,352 | 2,083 | 13,141 |
| Uterine cancer | 10,853 | 2,693 | 15,096 | 8,804 |
| Combined causes | 12,236 | 8,045 | 17,179 | 21,945 |
| Proportion per 100,000 alive in 1937 who died in 15 years from : | | | | |
| Breast cancer | 59 | 309 | 135 | 921 |
| Uterine cancer | 466 | 156 | 987 | 617 |
| Combined causes | 525 | 465 | 1,122 | 1,537 |

women lost 921 by breast and 617 by uterine cancer, or 1538 in all. The total expectation of dying of breast or uterine cancer during the next 30 years amongst 1000 women aged 30 must be about 20 in Japan and 22 in England and Wales, the lower risk from breast cancer being almost compensated by the higher risk from uterine cancer. Realisation of this must modify ideas about the effect of prolonged lactation on the incidence of breast cancer particularly in view of recent evidence that the important factor influencing the cervix cancer risk is early marriage rather than number of parturitions (Wynder *et al.*, 1954 ; Stocks, 1955).

*Mortality at different ages from mouth, oesophagus, intestine, larynx, and lung cancers, and leukaemia*

Table V compares the death rates in Japan in 1952 at various ages with the corresponding mean annual rates in England and Wales in 1952–54 for parts of the digestive tract, larynx and lung cancer, and leukaemia. Between ages 50 and 70 Japanese rates in men are about half the English rates for the buccal cavity and pharynx, and are about double the English rates for the oesophagus. For the intestine and rectum Japan's rates are about one-third of those in England and Wales for men between ages 40 and 60, and the ratio then decreases, whilst for women (as Table III shows) the ratio remains around 36 per cent from age 40 to 70. For the larynx the figures are similar in the two countries up to age 70, but for lung cancer registered mortality of men in Japan is less than one-tenth of the English rates at most ages, and amongst women the ratio is between one-half and one-third. Leukaemia rates change little with age in Japan, and they fail to show the increase from age 50 onwards which is such a pronounced feature of the English statistics.

### SUMMARY

Japanese mortality from malignant neoplasms as a whole ranks 9th for men and 15th for women amongst the equivalent average death rates at ages 35–64 of 21 countries when arranged in descending order. The statistics for separate forms of cancer up to age 70 may with safety be compared with those of other

TABLE V.—*Comparison between Age-specific Death Rates Per Million in Japan, 1952, and in England and Wales,* 1952–54 *from Cancer of Digestive and Respiratory Organs and Leukaemia*

| Age group. | Mouth and pharynx (140–8). Males. Japan. | Mouth and pharynx (140–8). Males. E. & W. | Oesophagus (150). Males. Japan. | Oesophagus (150). Males. E. & W. | Intestine and rectum (152–54). Males. Japan. | Intestine and rectum (152–54). Males. E. & W. | Larynx (161). Males. Japan. | Larynx (161). Males. E. & W. | Lung and bronchus (162–3). Males. Japan. | Lung and bronchus (162–3). Males. E. & W. |
|---|---|---|---|---|---|---|---|---|---|---|
| 0– . | 0·2 | 2 | 0·1 | 0 | 0·8 | 1·2 | 0·1 | 0·1 | 0·7 | 2·1 |
| 25– . | 2 | 2 | 1·2 | 1·2 | 7 | 17 | 0·5 | 0·2 | 5 | 26 |
| 35– . | 4 | 7 | 5 | 5 | 25 | 45 | 1·2 | 1·8 | 6 | 97 |
| 40– . | 9 | 11 | 15 | 11 | 38 | 91 | 6 | 5 | 21 | 249 |
| 45– . | 12 | 19 | 45 | 20 | 53 | 155 | 18 | 13 | 43 | 577 |
| 50– . | 22 | 47 | 95 | 53 | 109 | 284 | 32 | 33 | 70 | 1259 |
| 55– . | 40 | 92 | 208 | 99 | 176 | 511 | 57 | 75 | 114 | 2002 |
| 60– . | 72 | 148 | 362 | 170 | 262 | 854 | 85 | 101 | 181 | 2543 |
| 65– . | 78 | 170 | 550 | 277 | 423 | 1514 | 151 | 156 | 236 | 2922 |
| 70– . | *100* | 491 | *602* | 451 | *515* | 2443 | *146* | 225 | *219* | 2592 |
| 75– . | *131* | 799 | *587* | 699 | *521* | 3825 | *125* | 294 | *190* | 2072 |
| 80+ . | *97* | 1072 | *415* | 848 | *428* | 4719 | *83* | 357 | *110* | 1267 |

| Age group. | Leukaemia (204). Males. Japan. | Leukaemia (204). Males. E. & W. | Leukaemia (204). Females. Japan. | Leukaemia (204). Females. E. & W. | Intestine and rectum (152–54). Females. Japan. | Intestine and rectum (152–54). Females. E. & W. | Larynx (161). Females. Japan. | Larynx (161). Females. E. & W. | Lung and bronchus (162–3). Females. Japan. | Lung and bronchus (162–3). Females. E. & W. |
|---|---|---|---|---|---|---|---|---|---|---|
| 0– . | 20 | 33 | 14 | 25 | 0·5 | 0·9 | 0·1 | 0·1 | 0·7 | 0·6 |
| 25– . | 14 | 20 | 12 | 15 | 8 | 17 | 1·1 | 0·8 | 2·4 | 10 |
| 35– . | 20 | 27 | 16 | 24 | 20 | 45 | 0·8 | 2·2 | 3·5 | 26 |
| 40– . | 19 | 34 | 14 | 32 | 47 | 105 | 0·8 | 3·3 | 11 | 52 |
| 45– . | 16 | 44 | 15 | 33 | 63 | 174 | 8 | 7 | 18 | 89 |
| 50– . | 24 | 50 | 18 | 45 | 104 | 288 | 11 | 13 | 40 | 138 |
| 55– . | 19 | 80 | 13 | 62 | 184 | 469 | 15 | 17 | 40 | 201 |
| 60– . | 22 | 123 | 19 | 81 | 252 | 719 | 28 | 19 | 65 | 287 |
| 65– . | 20 | 162 | 18 | 113 | 377 | 1033 | 32 | 24 | 80 | 384 |
| 70– . | *16* | 169 | *12* | 126 | *386* | 1570 | *34* | 32 | *93* | 379 |
| 75– . | *3* | 211 | *19* | 136 | *426* | 2432 | *42* | 33 | *70* | 445 |
| 80+ . | *21* | 150 | *0* | 104 | *305* | 3667 | *33* | 39 | *18* | 362 |

countries having good registration and diagnostic arrangements. At ages after 70 certification of cancer as cause of death is probably less complete than in many countries of that class.

Comparison with England and Wales, and with Canada, shows that Japanese rates in middle life are high for cancer of the stomach, oesophagus and uterus, low for intestine, mouth and pharynx, breast and lung, and about the same for larynx. Despite the very low mortality from breast cancer, the total risk of dying from breast or uterine cancer between ages 30 and 60 is only slightly below that in England and Wales. Leukaemia rates are almost constant throughout life in Japan and do not show the increase after age 50 seen in English statistics.

It is suggested that a search for possible reasons for the low breast and high uterine cancer mortality, and for the low intestinal and high gastro-oesophageal cancer mortality in Japan might be helpful to the study of causation of these forms of cancer.

### REFERENCES

*Annual Epidemiological and Vital Statistics*, 1952.—(1955) World Health Organisation, Geneva.
*Mortality Trends in Canada*.—(1955) National Cancer Institute of Canada, Toronto.
MAISIN, J. H. AND LANGEROCK, G.—(1955) *Schweiz. Z. allg. Path.*, **18**, No. 4, 690.
SEGI, M., FUKUSHIMA, I., FUJISAKU, S., HONMA, H., MIKAMI, Y., KURIHAMA, M. AND SAITO, S.—(1955) *Cancer Mortality Statistics in Japan* 1900–1954. Tohoku University, Sendai.
SEGI, M.—(1955) *Schweiz. Z. allg. Path.*, **18**, No. 4, 668.
STOCKS, P.—(1955) *Brit. J. Cancer*, **9**, 487.
Transactions of 5th Meeting of Internat. Society of Geographical Pathology.—(1955) *Schweiz. Z. allg. Path.* **18**, No. 4, 385–950.
WYNDER E. L., CORNFIELD, J., SCHROFF, P. D. AND DORAISWAMI, K. R.—(1954) *Amer. J. Obstet. Gynec.*, **68**, 1016.